# Core-genome scaffold comparison reveals the prevalence that inversion events are associated with pairs of inverted repeats

**DOI:** 10.1186/s12864-017-3655-0

**Published:** 2017-03-29

**Authors:** Dan Wang, Shuaicheng Li, Fei Guo, Kang Ning, Lusheng Wang

**Affiliations:** 1 Department of Computer Science, City University of Hong Kong, 83 Tat Chee Ave., Hong Kong, SAR People’s Republic of China; 2 University of Hong Kong Shenzhen Research Institute, Shenzhen Hi-Tech Industrial Park, Nanshan District, Shenzhen, People’s Republic of China; 3 School of Computer Science and Technology, Tianjin University, Tianjin, People’s Republic of China; 4 School of Life Science and Technology, Huazhong University of Science and Technology, Wuhan, People’s Republic of China

**Keywords:** Comparative genomics, Genome rearrangment, Inversion, Transposition, Inverted block interchange

## Abstract

**Background:**

Genome rearrangement describes gross changes of chromosomal regions, plays an important role in evolutionary biology and has profound impacts on phenotype in organisms ranging from microbes to humans. With more and more complete genomes accomplished, lots of genomic comparisons have been conducted in order to find genome rearrangements and the mechanisms which underlie the rearrangement events. In our opinion, genomic comparison of different individuals/strains within the same species (pan-genome) is more helpful to reveal the mechanisms for genome rearrangements since genomes of the same species are much closer to each other.

**Results:**

We study the mechanism for inversion events via core-genome scaffold comparison of different strains within the same species. We focus on two kinds of bacteria, *Pseudomonas aeruginosa* and *Escherichia coli*, and investigate the inversion events among different strains of the same species. We find an interesting phenomenon that long (larger than 10,000 bp) inversion regions are flanked by a pair of Inverted Repeats (IRs). This mechanism can also explain why the breakpoint reuses for inversion events happen. We study the prevalence of the phenomenon and find that it is a major mechanism for inversions. The other observation is that for different rearrangement events such as transposition and inverted block interchange, the two ends of the swapped regions are also associated with repeats so that after the rearrangement operations the two ends of the swapped regions remain unchanged. To our knowledge, this is the first time such a phenomenon is reported for transposition event.

**Conclusions:**

In both *Pseudomonas aeruginosa* and *Escherichia coli* strains, IRs were found at the two ends of long sequence inversions. The two ends of the inversion remained unchanged before and after the inversion event. The existence of IRs can explain the breakpoint reuse phenomenon. We also observed that other rearrangement operations such as transposition, inverted transposition, and inverted block interchange, had repeats (not necessarily inverted) at the ends of each segment, where the ends remained unchanged before and after the rearrangement operations. This suggests that the conservation of ends could possibly be a popular phenomenon in many types of chromosome rearrangement events.

**Electronic supplementary material:**

The online version of this article (doi:10.1186/s12864-017-3655-0) contains supplementary material, which is available to authorized users.

## Background

Comparative genomics studies show that genome rearrangement events often occur between two genomes. Genome rearrangement events play important role in speciation. The rearrangement operations include deletions, insertions, inversion, transposition, block interchange, translocation, fission and fusion, etc. Here we study the mechanism for inversion events via core-genome scaffold comparison of different strains within the same species.

By comparing two genomes, we can find candidate rearrangement operations. However, the set of rearrangement operations to transform one genome into the other is not unique in many cases. Computing the rearrangement operations between two genomes under different assumptions is an active area, where intensive research have been conducted [1]. It is reported that breakpoints appear more often in repeated regions [2, 3]. A summary of the where and wherefore of evolutionary breakpoints is given by Sankoff [4]. The prevalence of short inversions has been studied [5]. Ranz et al. analyzed the breakpoint regions of the 29 inversions that differentiate the chromosomes of *Drosophila melanogaster* and two closely related species,*D. simulans* and *D. yakuba*, and reconstructed the molecular events that underlie their origin [6]. Rajaraman et al. suggested that rearrangements could be driven by the ISs and the positions of the inversion breakpoints in their study were also highly correlated with IS : 76 of the 118 mapped breakpoints were close (<1000 nt distant) to some predicted IS, whereas this number drops to 39 for uniformly sampled random coordinates (*P*-value <10^−3^) [7]. Darmon and Leach reviewed many examples of prokaryotic genomic rearrangements which were induced by natural transposable elements and pointed out that recombination between IRs can result in an inversion of the internal DNA sequence [8]. The association between IR and genome rearrangement breakpoints was also reported in previous studies on mammals and drosophila genomes [6, 9–12]. Armengol et al. observed that nine primary regions involved in human genomic disorders which show changes in the order or the orientation of mouse/human synteny segments were often flanked by segmental duplications in the human sequence [11]. They also found that 53% of all evolutionary rearrangement breakpoints associate with segmental duplications, as compared with 18% expected in a random location of breaks along the chromosome (*P*<10^−4^).

Pevzner and Tesler found extensive breakpoint reuse for inversion events in mammalian evolution when comparing human and mouse genomic sequences [13–15]. Statistics analyzes showed that breakpoints are often associated with repetitive elements and the density of breakpoints in small intergenes appears significantly higher than in gene deserts [3, 4, 16, 17]. Recently, breakpoint reuse for inversions has been reported in *Drosophila* genus [18, 19] as well as *Saccharomyces pastorianus* [20].

It is well known that recombination (crossing-over) of homologous or non-homologous DNAs can lead to various genetic variations including inversions, transpositions, insertions/deletions, and will leave some direct or inverted repeats on both ends, and existing repeats can further promote more variations.

To study the rearrangement operations, comparison of different individuals/strains within the same species (pan-genomes) can be more helpful since strains within the same species are conserved. A pan-genome, or supra-genome, describes the full complement of genes in a clade (typically for species in bacteria and archaea), which can have large variation in gene content among closely related strains. Pan-genomes were first studied by Tettelin more than a decade ago [21]. Several tools have been developed for pan-genome analysis. For example, GET_HOMOLOGUES [22] is a customizable and detailed pan-genome analysis platform. BLAST atlas [23] visualizes which genes from the reference genome are present in other genomes. Mugsy-Annotator [24] identifies syntenic orthologs and evaluates annotation quality using multiple whole genome alignments. Characterization of the core and accessory genomes of *Pseudomonas aeruginosa* has been done by Ozer et al. [25]. For pan-genome analysis, genomes from different strains of the same species are decomposed to core blocks (shared by all the genomes), dispensable blocks (shared by a subset of the genomes) and strain-specific blocks (unique to a single genome). Here we extend the pan-genome analysis by comparing the core-genome scaffolds of different strains of the same species.

We study two types of bacteria, *Pseudomonas aeruginosa* and *Escherichia coli*, and investigate the inversion events among different strains of the same species. We find an interesting phenomenon that long (larger than 10,000 bp) inversion regions are flanked by pairs of Inverted Repeats (IRs) which are often Insertion Sequences (ISs). This mechanism also explains why the breakpoint reuses for inversion events happen. We study the prevalence of the phenomenon and find that it is a major mechanism for inversions. The other observation is that for different rearrangement events such as transposition and inverted block interchange, the two ends of the swapped regions are also associated with repeats so that after the rearrangement operations the two ends of the swapped regions remain unchanged. To our knowledge, this is the first time such a phenomenon is reported for transposition event.

## Methods

We develop a pipeline to generate the core-genome blocks, dispensable blocks and strain-specific blocks based on the multiple sequence alignment produced by Mugsy [26].

We then develop a computer program to generate the scaffolds of the strains from the core-genome blocks by repeatedly merging two consecutive blocks appearing in all the strains of the same species. In this way, the number of distinct blocks in the core-genome scaffold is reduced dramatically. For example, for *Pseudomonas aeruginosa*, before merging, there are 185 blocks in the core genome of the 25 strains. After merging, the scaffolds contain 69 blocks.

After that, we compute the inversion distance between two scaffolds. Computing the inversion distance between two scaffolds is a very hard and complicated combinatorial problem. Several algorithms have been developed. Due to the difficulty of algorithm design, most of the algorithms only consider inversion events. However, a transposition/block-interchange event can be represented as 3 inversion events, and an inverted transposition/block-interchange event can be represented as 2 inversion events. Therefore, some of the computed inversion events may not be real. There are algorithms dealing with inversion and other rearrangement events such as block interchanges simultaneously. However, the weights for different events are different (again due to the difficulty of algorithm design). Thus, those algorithms still suffer from the problem of outputting inversions that are not real.

Our strategy here is to eliminate some obvious (independent) transposition, inverted transposition, block interchange, and inverted block interchange events before computing the inversion distance between two scaffolds.

For simplicity, we always assume that *G*_1_=+1+2…+*n* is the first input scaffold and *G*_2_=*π*_1_ *π*_2_…*π*_*n*_ is a sign permutation of the *n* blocks over the set *N*={1,2,…,*n*} of *n* distinct blocks, where each integer *i*∈*N* appear once in *G*_2_ in the form of either +*i* or −*i*. All the rearrangement operations are on *G*_2_.

A *transposition* swaps the order of two consecutive blocks/regions without changing their signs. A transposition (*i*,*j*,*k*) on regions *π*_*i*_,…,*π*_*j*−1_ and *π*_*j*_…*π*_*k*−1_ transforms the sign permutation *π*_1_…*π*_*i*−1_ *π*_**i**_…*π*_**j**−**1**_ *π*_**j**_…*π*_**k**−**1**_ *π*_*k*_…*π*_*n*_ into *π*_1_…*π*_*i*−1_ *π*_**j**_…*π*_**k**−**1**_ *π*_**i**_…*π*_**j**−**1**_ *π*_*k*_…*π*_*n*_.

**Fig. 1:**
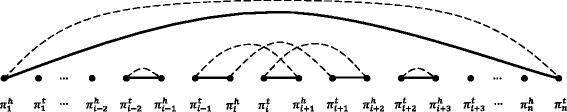
The breakpoint graph for an independent transposition

A transposition is *independent* if it transforms the sign permutation *π*_1_…*π*_*i*−2_ *π*_*i*−1_ *π*_**i**+**1**_ *π*_**i**_ *π*_*i*+2_ *π*_*i*+3_…*π*_*n*_ into *π*_1_…*π*_*i*−2_ *π*_*i*−1_ *π*_**i**_ *π*_**i**+**1**_ *π*_*i*+2_ *π*_*i*+3_…*π*_*n*_, where *π*_*i*−1_ *π*_**i**_ *π*_**i**_+**1** *π*_*i*+2_ is either +(*q*−1)+*q*+(*q*+1)+(*q*+2) or −(*q*+2)−(*q*+1)−*q*−(*q*−1) for {*q*−1,*q*,*q*+1,*q*+2}⊆*N*={1,2,…,*n*}. Though an independent transposition swaps two consecutive blocks *π*_**i**+**1**_ and *π*_**i**_ instead of two regions *π*_*i*_,…,*π*_*j*−1_ and *π*_*j*_…*π*_*k*−1_ as in the definition of a general transposition, a pre-process allows us to merge two consecutive blocks if they are consecutive in both input genomes. Thus, we can still handle some cases for swapping two consecutive regions. For example, the genome +1+2+6+7+3+4+5+8 becomes +1+2+**4**+**3**+5 after merging +6+7 (represented as +**4**)and +3+4+5 (represented as +**3**) and re-number +8 as +5 in the new representation. An independent transposition can change +1+2+**4**+**3**+5 into +1+2+3+4+5. In terms of breakpoint graph, the two blocks *π*_**i**+**1**_ *π*_**i**_ in an independent transposition is involved in a 6-edge cycle and after the transformation the 6-edge cycle becomes three 2-edge cycles. In other words, the three breakpoints involved in the 6-edge cycle disappear after the transformation. See Fig. 1.

An *inverted transposition* swaps the order of two consecutive blocks/regions with one of the block’s sign changed. An inverted transposition (*i*,*j*,*k*) on regions *π*_*i*_,…,*π*_*j*−1_ and *π*_*j*_…*π*_*k*−1_ transforms the sign permutation *π*_1_…*π*_*i*−1_ *π*_**i**_…*π*_**j**−**1**_ *π*_**j**_…*π*_**k**−**1**_ *π*_*k*_…*π*_*n*_ into *π*_1_…*π*_*i*−1_−*π*_**k**−**1**_…−*π*_**j**_ *π*_*i*_…*π*_**j**−**1**_ *π*_*k*_…*π*_*n*_ or *π*_1_…*π*_*i*−1_ *π*_**j**_…*π*_**k**−**1**_−*π*_**j**−**1**_…−*π*_**i**_ *π*_*k*_…*π*_*n*_.

An inverted transposition is *independent* if it transforms the sign permutation *π*_1_…*π*_*i*−2_ *π*_*i*−1_−*π*_**i**+**1**_ *π*_**i**_ *π*_*i*+2_ *π*_*i*+3_…*π*_*n*_ or *π*_1_…*π*_*i*−2_ *π*_*i*−1_ *π*_**i**+**1**_−*π*_**i**_ *π*_*i*+2_ *π*_*i*+3_…*π*_*n*_ into *π*_1_…*π*_*i*−2_ *π*_*i*−1_ *π*_**i**_ *π*_**i**+**1**_ *π*_*i*+2_ *π*_*i*+3_…*π*_*n*_, where *π*_*i*−1_ *π*_**i**_ *π*_**i**+**1**_ *π*_*i*+2_ is either +(*q*−1)+*q*+(*q*+1)+(*q*+2) or −(*q*+2)−(*q*+1)−*q*−(*q*−1) for {*q*_1_,*q*,*q*+1,*q*+2}⊆*N*={1,2,…,*n*}.

A *block interchange* swaps the locations of two separated blocks without changing their signs. A block interchange (*i*,*j*,*k*,*l*) on regions *π*_*i*_…*π*_*j*_ and *π*_*k*_…*π*_*l*_ transforms *π*_1_…*π*_*i*−1_ *π*_**k**_…*π*_**l**_ *π*_*j*+1_…*π*_*k*−1_ *π*_**i**_…*π*_**j**_ *π*_*l*+1_ … *π*_*n*_ into *π*_1_…*π*_*i*−1_ *π*_**i**_…*π*_**j**_ *π*_*j*+1_…*π*_*k*−1_ *π*_**k**_…*π*_**l**_ *π*_*l*+1_…*π*_*n*_.

**Fig. 2:**
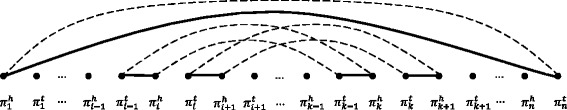
The breakpoint graph for an independent block interchange

A block interchange is *independent* if it transforms the sign permutation *π*_1_…*π*_*i*−1_ *π*_**k**_ *π*_*i*+1_…*π*_*k*−1_ *π*_**i**_ *π*_*k*+1_…*π*_*n*_ into *π*_1_…*π*_*i*−1_ *π*_**i**_ *π*_*i*+1_…*π*_*k*−1_ *π*_**k**_ *π*_*k*+1_…*π*_*n*_, where *π*_*i*−1_ *π*_**i**_ *π*_*i*+1_ is either +*q*+(*q*+1)+(*q*+2) or −(*q*+2)−(*q*+1)−*q* and *π*_*k*−1_ *π*_**k**_ *π*_*k*+1_ is either +*p*+(*p*+1)+(*p*+2) or −(*p*+2)−(*p*+1)−*p* for {*q*,*q*+1,*q*+2}⊆*N* and {*p*,*p*+1,*p*+2}⊆*N*. Similarly, the two blocks *π*_*k*_ and *π*_*i*_ are involved in two (interleaving) 4-edge cycles in the breakpoint graph and after the transformation, they become four 2-edge cycles. In other words, there are four breakpoints at the two ends of the two blocks, after the transformation, the four breakpoints disappear. See Fig. 2.

An *inverted block interchange* swaps the location of two separated blocks with both signs of the two blocks changed. An inverted block interchange (*i*,*j*,*k*,*l*) on regions *π*_*i*_…*π*_*j*_ and *π*_*k*_…*π*_*l*_ transforms *π*_1_…*π*_*i*−1_−*π*_**l**_…−*π*_**k**_ *π*_*j*+1_…*π*_*k*−1_−*π*_**j**_…−*π*_**i**_ *π*_*l*+1_…*π*_*n*_ into *π*_1_…*π*_*i*−1_ *π*_**i**_…*π*_**j**_ *π*_*j*+1_…*π*_*k*−1_ *π*_**k**_…*π*_**l**_ *π*_*l*+1_…*π*_*n*_.

An inverted block interchange is *independent* if it transforms the sign permutation *π*_1_…*π*_*i*−1_−*π*_**k**_ *π*_*i*+1_…*π*_*k*−1_−*π*_**i**_ *π*_*k*+1_…*π*_*n*_ into *π*_1_…*π*_*i*−1_ *π*_**i**_ *π*_*i*+1_…*π*_*k*−1_ *π*_**k**_ *π*_*k*+1_…*π*_*n*_, where *π*_*i*−1_ *π*_**i**_ *π*_*i*+1_ is either +*q*+(*q*+1)+(*q*+2) or −(*q*+2)−(*q*+1)−*q* and *π*_*k*−1_ *π*_**k**_ *π*_*k*+1_ is either +*p*+(*p*+1)+(*p*+2) or −(*p*+2)−(*p*+1)−*p* for {*q*,*q*+1,*q*+2}⊆*N* and {*p*,*p*+1,*p*+2}⊆*N*. Again, there are four breakpoints at the two ends of the two blocks −*π*_*i*_ and −*π*_*k*_, after the transformation, the four breakpoints disappear.

After eliminating independent transposition, inverted transposition, block interchange and inverted block interchange events, we use GRIMM-Synteny [27, 28] to compute the inversion distance between pairwise core-genome scaffolds. We only seriously consider the cases where the rearrangement distance is small. When the rearrangement distance is large, there may be multiple solutions for the inversion history. Thus, in this case, the computed inversion events may not be real.

Finally, we developed a pipeline to compare sequences at the two ends of each inversion region to see whether a pair of inverted repeats exists. Once the inverted repeats are found, the pipeline can also search all the strains and mark down its positions in different strains.

## Results

### *Pseudomonas aeruginosa*

**Fig. 3:**
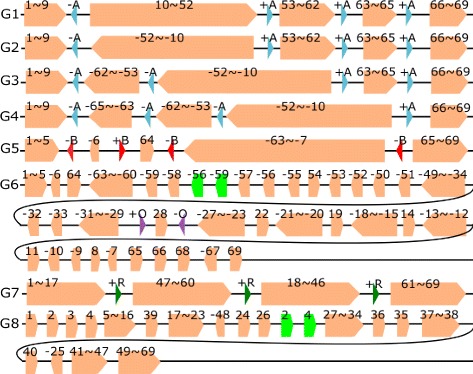
Eight groups of scaffolds for the 25 *Pseudomonas aeruginosa* strains. Each *orange block* stands for a merged block which may represent several consecutive core-genome blocks. The numbers above each *orange block* indicate the included core-genome blocks, for example, 1$\thicksim $5 means the *orange block* includes five core-genome blocks, which are Blocks 1, 2, 3, 4 and 5. Repeats A, B, O and R are represented by *blue*, *red*, *purple and green triangles* respectively. The arrow directions indicate positive/negative strand

Complete genome sequences of 25 *Pseudomonas aeruginosa* strains PACS2, F22031,NCGM1900, LES431, NCGM2.S1, Carb01_63, SCV20265, UCBPP-PA14, VRFPA04, DSM_50071, 19BR, 213BR, B136-33, PA7, PA1, YL84, LESB58, M18, RP73, DK2, MTB1, PAO1, PA1R, NCGM1984,and FRD1 were downloaded from NCBI GenBank. The details of these 25 *Pseudomonas aeruginosa* strains are listed in Additional file 1: Table S1. The genome lengths of these strains are between 6.2 mbp (million base pair) and 7.5 mbp. We used our pipeline to compute the core-genomes and obtained 533 core-blocks with lengths ranging from 58 bp to 83 kbp (kilo base pair) and total lengths ranging from 5.33 to 5.6 mbp (million base pair) which account for 74.8–88.2% of the strains’ genomes. We then eliminated core blocks with length less than 500 bp and iteratively merged core blocks that were consecutive for all the 25 strains. As a result, 69 (merged) blocks were obtained and the 25 strains led to 8 different scaffolds as shown in Fig. 3. The scaffold for each *Pseudomonas aeruginosa* strain is in Additional file 1: Table S4. For any pair of consecutive blocks in one group, there must be a different group in which there is a breakpoint between the two blocks when comparing the two scaffolds.

Group 1 contains 13 strains, which are *Pseudomonas aeruginosa* strains NCGM1984, B136-33, YL84, M18, LESB58, SCV20265, LES431, UCBPP-PA14, DK2, MTB-1, DSM_50071, Carb01_63, and F22031. Group 2 contains 6 strains, which are strains RP73, 213BR, PA1, PA1R, 19BR, and PAO1. Groups 3-8 contain 1 strain each and the respective strains are PACS2, FRD1, NCGM2.S1, VRFPA04, NCGM1900, and PA7.

We computed the pairwise inversion distance between scaffolds after eliminating other kinds of independent rearrangement events such as transpositions, inverted-transpositions, block-interchanges, and inverted-block-interchanges. For each of the 8 scaffolds, we chose a scaffold with the minimum inversion distance (after eliminating other independent rearrangement events) to compare. The purpose was to compare two scaffolds with a small number of inversions so that we can observed real inversions between them. From Table 1, it can be seen that Group 1 is the closest group to all the other groups except for Group 6. The closest group to Group 6 is Group 5, where the inversion distance is 7.

**Table 1:** Shortest inversion distance for each of the 8 groups of *Pseudomonas aeruginosa*

*sG* ^a^	*cG* ^a^	*I* *n* *v*_*d*_^b^	*inversion* ^c^	*l* ^d^	*IR* ^e^	*R*_*d*_^b^
1	2	1	(10,52)	4.061	A(2)	0
2	1	1	(-52,-10)	4.061	A(2)	0
3	1	1	(-62,-10)	4.769	A(2)	0
4	1	1	(-65,-10)	5.699	A(2)	0
5	1	3	(-6,-6)	0.0597	B(0)	0
			(64,64)	0.0068	B(0)	
			(-64,-7)	5.879	B(0)	
6	5	7	(28,28)	0.0130	O(0)	3
			(54,54)	0.0025	None	
			(22,22)	0.0032	None	
			(19,19)	0.0031	None	
			(14,14)	0.0021	None	
			(11,11)	0.0054	None	
			(8,8)	0.0074	None	
7	1	0	None	N/A	N/A	1
8	1	0	None	N/A	N/A	4

^a^Column sG is the source scaffold group, Column cG is the closest scaffold group

^b^ *I* *n* *v*_*d*_ indicates the inversion distance between sG and cG after eliminating other independent rearrangement events. *R*_*d*_ indicates the distance of other independent rearrangement events

^c^The two numbers indicate the starting and ending block of the inversion in the source scaffold (sG). Rearrangement scenario is calculated from the source group to the closest group

^d^ *l* is the length (in mbp) of inversion of the core-genome segments

^e^Column *IR* lists which pair of inverted repeats (A, B or O) flanks the inversion. The numeric code: 0 indicates the respective IR was found only in the source group, 1 indicates the IR was found only in the closest group, 2 indicates the IR was found in both groups

In total, there are 13 inversion events among the 7 distinct pairs of scaffolds (Table 1, where pair 1 and 2 appears twice). Among the 13 inversion regions, 7 of them are flanked by a pair of IRs. The remaining 6 inversions with no IRs found at the two ends of the inversion regions are very short and their lengths are from 2100 to 7400 bp. For each of the first three (Table 1, rows 1-4) inversions, the lengths of the inversion regions are more than 4 mbp, and we find a pair of IRs (+A/-A) at the two ends of each of the three long inversion regions. For the pair of Groups 5 and 1, there are three inversions and the lengths of the three inversions in the core-genome are 5.879 mbp, 0.597 mbp, and 6.8 kbp, respectively. Interestingly, we find a repeat *B* that appears four times in Both Scaffold 1 and Scaffold 5, where *B* appear as −*B* once and as +*B* three times in Scaffold 1. The four occurrences of *B* form a pair of IRs at the two ends of each of the 3 inversion regions (see Fig. 3). For Groups 6 and 5, there exist two independent transpositions and one inverted transposition (see Additional file 2). After eliminating the three independent rearrangement events, there are 7 inversions between Groups 6 and 5 which are calculated by GRIMM-Synteny (see Additional file 2) and only one inversion (28,28) is flanked by a pair of IRs (see Table 1). Note that both −56 and −59 appear twice in Scaffold 6. We remove the green blocks in Fig. 3 in our comparison. Among these seven inversions, only one inversion (28,28) is longer than 10000 bp and flanked by a pair of IRs (+O/-O). Group 1 can be obtained from Group 7 with one independent transposition. A repeat +R appears three times at the ends of the two blocks involved in the transposition. See Fig. 3). Those occurrences of +R play an important role in the transposition and the details will be discussed in the Transposition section. For Group 8 and 1, there exist two independent transpositions and two independent inverted transpositions (see Additional file 2). After eliminating the four independent rearrangement events, the scaffolds for Group 8 and 1 are actually the same and the inversion distance between them is zero. Again, both Blocks 2 and Block 4 appear twice in Group 8. (The physical positions of all the copies of Blocks 2 and 4 in Group 8 are in Additional file 1: Table S5h). We remove the green blocks in Fig. 3 in our comparison.

For the first inversion between Group 1 and 2, there are 13 strains in Group 1 and 6 strains in Group 2. All the strains in Group 1 and Group 2 contain Repeat +*A* and −*A* as shown in Fig. 3. The physical positions as well as the lengths of the repeats differ slightly in different strains. See Additional file 1: Table S5a. Thus, the inversion (from Blocks 10 to 52) between Scaffold 1 and Scaffold 2 (row 1 in Table 1) is found between the 13×6 pairs of strains in these two groups. For the remaining inversions listed in Table 1, the physical positions, the lengths of repeats and core-genome blocks (at the two ends of an inversion) in different strains are given in Additional file 1: Tables S5b-e.

In summary, three different pairs of IRs are found and we use +A/-A, +B/-B and +O/-O to differentiate these three pairs. We also find three copies of +R in comparison of Groups 1 and 7. The locations of these repeats in the scaffolds are shown in Fig. 3. The lengths (in bp), gene products and protein IDs (in NCBI Protein database) of these repeats are listed in Additional file 1: Table S3.

#### Breakpoint reuse

**Fig. 4:**
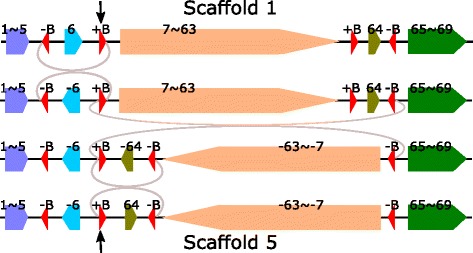
Three inversion steps from scaffold 1 to scaffold 5. The breakpoint between -6 and 64 in Scaffold 5 is used three times. See the *black arrow*

The three inversion steps from Scaffold 1 to 5 are shown in Fig. 4, where it can be seen that there is a +B and three -Bs in Scaffold 5. The three inversion events are -B-6+B to -B6+B, +B7$\thicksim $64-B to +B-64$\thicksim $-7-B and +B-64-B to +B64-B and the breakpoint the black arrow points at in Fig. 4 is used three times.

Here +B plays a crucial role in the three inversions and is used three times, each time +B and -B form a pair of inverted repeats at the two ends of the inversion regions. Now let us have a close look at +B (of length 820 bp), we can see that for the first inversion (-B-6+B to -B6+B), the real cutting points (breakpoints) are at the left end of -B and the right end of +B, while for the other two inversions (+B7$\thicksim $64-B to +B-64$\thicksim $-7-B and +B-64-B to +B+64-B), the real cutting points (breakpoints) are at the left end of +B and the right end of -B. Here the real cutting point does not seem to be important and the repetitive element B should be viewed as the breakpoint.

Another interesting finding is that for Groups 1, 2, 3 and 4, each scaffold contains a -A and three +As. (See Fig. 3.) Theoretically, this -A can be reused three times with each of the three +As. However, we did not observe such three breakpoint reuses in a single pairwise scaffold comparison. But it has been observed that this -A, along with each of the three +As, mediate three different inversion events which occur between Group 1 and Group 2, Group 1 and Group 3, and Group 1 and Group 4, respectively (Table 1, row 2-4).

#### Transposition

**Fig. 5:**
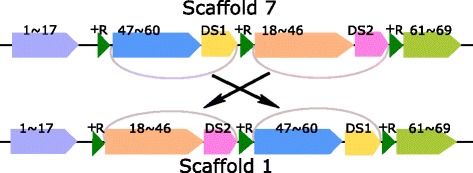
The role of repeats in transposition event. Both Scaffolds 1 and 7 contain four merged core blocks (1$\thicksim $17), (18$\thicksim $46), (47$\thicksim $60), and (61$\thicksim $69). Moreover, both Scaffolds 1 and 7 contain another two non-core blocks DS1 and DS2, where the occurrences of DS1 and DS2 in both scaffolds are 100% identical. There are three occurrences of a repeat +R in both scaffolds

Figure 5 gives the detailed scaffolds for Groups 1 and 7. Both Scaffolds 1 and 7 contain four merged core blocks (1$\thicksim $17), (18$\thicksim $46), (47$\thicksim $60), and (61$\thicksim $69). Moreover, both Scaffolds 1 and 7 contain another two non-core blocks DS1 and DS2, where the occurrences of DS1 and DS2 in both scaffolds are 100% identical. Besides, there are three occurrences of a repeat +R in both scaffolds. It can be seen that by swapping 47$\thicksim $60 and DS1 with 18$\thicksim $46 and DS2, Scaffold 7 is transferred into Scaffold 1. The most interesting finding is the three occurrences of +R located at the three breakpoints of the transposition. We believe that this three occurrences of +R play an important role in this transposition event because the repeat +R can make sure the two ends of the two swapped regions remain unchanged before and after the transposition. This is similar to the mechanism that inversion regions are franked by a pair of IRs, where after the inversion the two ends of the inversion region remain the same. For reference, the physical positions of the three +Rs, DS1, DS2 and Blocks 47, 60, 18 and 46 in the chromosomes of Group 7 and 1 are listed in Additional file 1: Table S5f. To our knowledge, this is the first example that three copies of a repeat were found at the two ends of the two swapped segments in transposition.

### *Escherichia coli*

**Fig. 6:**
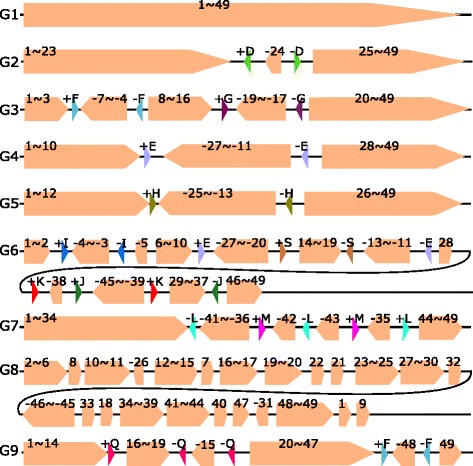
Nine groups of scaffolds for the 31 *Escherichia coli* strains

We selected 31 *Escherichia coli* strains (identification number (id) 1 to 31) with complete sequences from 17 genome families at NCBI’s GenBank. These 31 strains are SE15, IAI39, EC4115, CFT073, CE10, O103:H2 str. 12009, C227-11, 536, K-12 substr. MG1655, ST2747, NA114, 042, O111:H- str. 11128, O145:H28str.RM13514, O104:H4 str. 2011C-3493, SE11, SS52, APEC O78, SMS-3-5, DH1Ec095, 1303, O157:H7 str. Sakai, 55989, B str. REL606, O83:H1 str. NRG 857C, UMN026, PCN033, 789, O127:H6 str. E2348/69, P12b, and ED1a. The detailed information of these 31 strains is listed in Additional file 1: Table S2. The genome lengths of these strains are between 4614223 bp and 5585613 bp. Our pipeline found 344 core blocks. The lengths of these core blocks range from 45 to 72931 bp and the total core-genome lengths in different strains range from 4006932 to 4246034 bp which account for 74.07–88.42% of the strains’ genomes. After eliminating core-blocks with length less than 500 bp and repeatedly merge two consecutive core-blocks (that are consecutive for all the 31 strains), we obtained 49 (merged) blocks and the 31 strains formed 9 groups of scaffolds (G1-G9 as shown in Fig. 6). The scaffold for each of the 31 *Escherichia coli* strain is given in Additional file 1: Table S6.

Group 1 contains 21 strains which are *Escherichia coli* strains EC4115, CE10, C227-11, K-12 substr. MG1655, ST2747, 042 O104:H4 str. 2011C-3493, SE11, SS52, APEC O78, DH1Ec095, 1303, O157:H7 str. Sakai, 55989, B str. REL606, O83:H1 str. NRG 857C, UMN026, PCN033, 789, O127:H6 str. E2348/69, and ED1a. Group 2 contains 3 strains, SE15, CFT073 and 536. Groups 3-9 contain 1 strain each and the respective strains are O145:H28 str. RM13514, SMS-3-5, P12b, IAI39, O103:H2 str. 12009, NA114, and O111:H- str. 11128.

After computing pairwise inversion distance among the 9 scaffolds, we selected a scaffold with minimum inversion distance for each of the 9 scaffolds as shown in Table 2 for comparison. From Table 2, it can be seen that Group 1 is the closest group to all the other 8 groups with inversion distances ranging from 0 to 4. The closest group to Group 1 is Group 2, where the sign of Block 24 is different.

**Table 2:** Shortest inversion distannce for each of the 9 groups of *Escherichia coli*

*sG*	*cG*	*I* *n* *v*_*d*_	*inversion*	*l*	*IR*	*R*_*d*_
1	2	1	(24,24)	0.0041	D(1)^a^	0
2	1	1	(-24,-24)	0.0041	D(0)^a^	0
3	1	2	(-7,-4)	0.2763	F(0)	0
			(-19,-17)	0.1940	G(0)^b^	
4	1	1	(-27,-11)	1.402	E(0)	0
5	1	1	(-25,-13)	1.111	H(0)	0
6	1	4	(-4,-3)	0.2706	I(0)	1
			(-5,-5)	0.0075	–	
			(-45,37)	1.4108	J(0)	
			(-38,-29)	1.2756^c^	K(0)	
7	1	3	(-43,-35)	0.0642	L(0)	0
			(-42,35)	0.1055^d^	M(0)	
			(-41,-35)	0.3944^e^	L(0)	
8	1	4	(In Additional	N/A	N/A	6
			file 2)			
9	1	1	(48,48)	0.0651	F(0)	1

^a^In Group 1, only Strain SE15 has +D/-D at the ends of 24

^b^In Group 2, only Strain O157:H7 str. Sakai has +G/-G at the ends of (-19,-17)

^c^ *l*=length of Block 38 + length from Block 29 to Block 37 in Group 6

^d^ *l*=length of Block 42 + length of Block 35 in Group 7

^e^ *l*=length from Block 41 to Block 36 + length of Block 35 in Group 7

In total, there are 17 inversion events among the 8 distinct pairs in Table 2 (the pair of Group 1 and Group 2 appears twice) and the inversion region lengths varies from 0.0075 to 1.402 mbp. (See Table 2.) Among the 17 inversion regions, 12 of them are found to be flanked by a pair of inverted repeats in the strains of the source groups. For inversion (-5,5) between Group 1 and Group 6 (row 6 in Table 2) and the four inversions between Group 1 and 8, no pairs of inverted repeats are found at the two ends of the block. The length of inversion (-5,5) (Row 6 in Table 2) is short (7.5 kbp). The four computed inversions between Groups 1 and 8 may not be true since there are another 6 other rearrangement events between the two scaffolds (Row 8 in Table 2). For Groups 6 and 1, the rearrangement distance is five (one independent inverted block interchange and a sequence of four inversions). See Table 2. At the breakpoints of this inverted block interchange, we also find IRs and we will discuss it later in the Inverted Block Interchange section. For Group 8 and 1, after eliminating six independent transpositions, there exists a sequence of four inversions (see Additional file 2). Only one of these four inversions is flanked by a pair of IRs. We observe that there are seven copies of Block 45 in Group 8 and we used the -45 next to -46 for comparison. The distance between Group 1 and Group 8 is big (6 transpositions + 4 inversions) and thus our predicted rearrangement history between Group 1 and Group 8 may not be correct. (Again, for reference, the physical positions of these seven copies of Block 45 in the chromosome of Group 8 are in Additional file 1: Table S7i.) To obtain Group 1 from Group 9, an independent inverted transposition and an inversion (Block -48 in Scaffold 9) are required. (See Table 2). The inverted region (Block -48) is flanked by a pair of IRs (+F/-F) in the Group 9. (See Fig. 6.) In addition, we find that this inverted transposition event is also associated with repeats and we will discuss this later in the “Inverted transposition” section.

For all the inversions listed in Table 2, the physical positions, the lengths of repeats and core-genome blocks (at the two ends of inversions) in different strains are given in Additional file 1: Table S7a-g.

We find a total of 12 different types of pairs of inverted repeats and use letters from +D/-D to +M/-M, +S/-S and +Q/-Q to label and differentiate these 12 pairs of IRs. The locations of these IRs in the scaffolds are shown in Fig. 6. The lengths (in bp), gene products and protein IDs (in NCBI Protein database) of these 12 IRs are listed in Additional file 1: Table S8. We note that 7 of these 12 pairs of IRs contain genes which encode transposase.

#### Breakpoint reuse

**Fig. 7:**
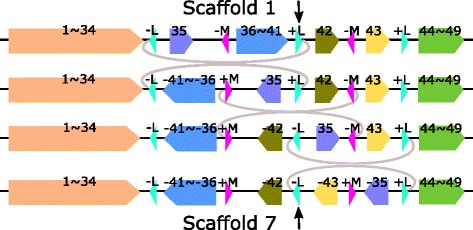
Three inversions between Scaffolds 1 and 7. The breakpoint between 41 and 42 in Scaffold 1 is used twice. See the *black arrow*

The three inversion steps from Scaffolds 1 to 7 are illustrated in Fig. 7. From Fig. 7, it can be seen that The breakpoint between 41 and 42 in Scaffold 1 is used twice. The corresponding inversion regions are flanked by -L and +L.

It is worth pointing out that the two +Ms in Scaffold 1 form a pair of directed repeats (DRs). After inversion (35,-41), the pair of directed repeats (DRs) of M becomes a pair of inverted repeats. This means that a pair of DRs has the potential to mediate inversions.

#### Inverted block interchange

**Fig. 8:**
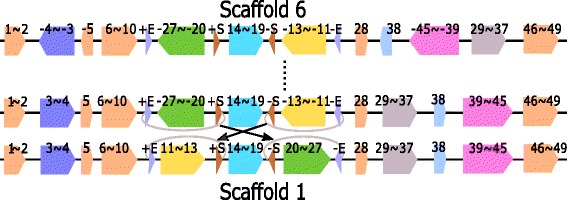
Inverted block interchange of Region -27$\thicksim $-20 and Region -13$\thicksim $-11 between Scaffolds 6 and 1. +E/-E and +S/-S are two pairs of IRs. The steps from Scaffold 6 to the middle scaffold are omitted

We find an inverted block interchange between Scaffold 6 and 1 and we use Fig. 8 to illustrate. In Fig. 8, Region +E-27$\thicksim $-20+S and –S13$\thicksim $-11-E in Scaffold 6 are inversely interchanged with each other to obtained Scaffold 1. The existence of two pairs of IRs (+E/-E and +S/-S) makes sure the two ends of the swapped blocks remain unchanged after the inverted block interchange event. The physical positions of +E/-E, +S/-S and Blocks 27, 20, 13 and 11 in Groups 6 and 1 are listed in Additional file 1: Table S7h.

**Fig. 9:**
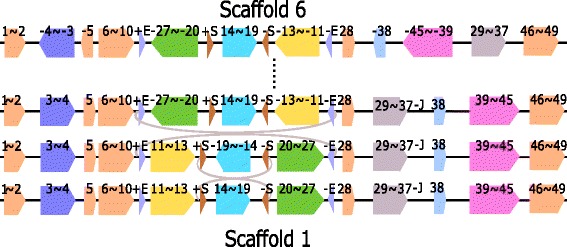
Two inversions which can replace the inverted block interchange of Regions -27$\thicksim $-20 and -13$ \thicksim $-11 between Scaffold 6 and 1. The first inversion is flanked by +E and -E and the second inversion is flanked by +S and -S. The steps from the Scaffold 6 to its next scaffold are omitted

The other explanation is that an inverted block interchange can be replaced by two inversions. Figure 9 shows the two inversions which can replace the inverted block interchange of Blocks -27$\thicksim $-20 and Block -13$\thicksim $-11. Each of these two inversions is flanked by a pair of IRs (see Fig. 9).

#### Inverted transposition

**Fig. 10:**
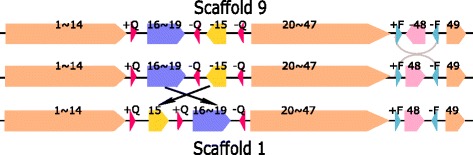
Inverted transposition of Region 16$\thicksim $19 and Block 15 between Scaffold 9 and 1. There are three occurrences of Repeat Q with different signs. From Scaffold 9 to the next scaffold, there is an inversion of Block 48 which are flanked by +F and -F

Figure 10 shows the inverted transposition from Scaffolds 9 to 1: Block -15 and region 16$\thicksim $18 in Scaffold 9 are swapped with each other with the sign of Block -15 changed. Block -15 is flanked by a pair of directed repeats (DRs) (-Q,-Q) and Region 16$\thicksim $18 is flanked by a pair of IRs (+Q,-Q) in Scaffold 9. These three occurrences of Repeat Q can make the ends of Block -15 and Block 16-18 remain unchanged after the inverted transposition (with the sign of Block -15 changed). The physical positions of the three copies of Repeat Q and Blocks 15, 16 and 18 in the chromosomes of Group 9 and 1 are listed in Additional file 1: Table S7j.

**Fig. 11:**
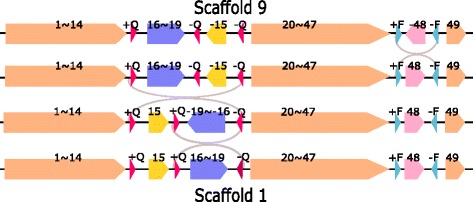
Two inversions which can replace the inverted transposition of Region 16$\thicksim $19 and Block 15 between Scaffold 9 and 1. Both of the two inversions are flanked by a pair of IRs (+Q/-Q). From Scaffold 9 to the next scaffold, there is an inversion of Block 48 which are flanked by +F and -F

The other explanation is that the inverted transposition can be replaced by two inversions: the first inversion is from Blocks 16 to -15 and the second inversion is from Blocks -19 to -16 (see Fig. 11). Both of these two inversions are flanked by a pair of IRs (+Q/-Q).

## Discussion

For both *Pseudomonas aeruginosa* and *Escherichia coli* strains, IRs were found at the two ends of long sequence inversions. The two ends of the inversion remained unchanged before and after the inversion event. We also observed that other rearrangement operations such as transposition, inverted transposition, and inverted block interchange, had repeats (not necessarily inverted) at the ends of each segment, where the ends remained unchanged before and after the rearrangement operations. This suggests that the conservation of ends could possibly be a popular phenomenon in many types of chromosome rearrangement events. Past studies reveal that insertions and deletions (indels) can be mediated by directed repeats (DRs) [29, 30]. Sequences flanked by a pair of DRs can be deleted from a chromosome and the resulting chromosomes keeps only one copy of the DR. Sequences flanked by a pair of DRs can also be inserted into a chromosome at the location containing one copy of the DR. In both case, the ends of the indels remain unchanged [29, 30]. However, indels can also be caused by activities of mobile DNA elements such as transposons and integrons, where conservation of ends does not hold [31].

The mechanism for breakpoint reuse is also interesting. The fact that long inversions are flanked by a pair of inverted repetitive elements can clearly explain why breakpoint reuse happens for inversions. Our observations show that the breakpoint reuse is actually the repeated segment reuse. The breakpoints at the nucleotides level for the reused repeat differ depending on the repeat is at the left or right end of the inversion.

## Conclusions

In this paper, we have studied the rearrangement events for both *Pseudomonas aeruginosa* and *Escherichia coli* strains. We have found that repeats were at the ends of different kinds of rearrangement events including inversion, transposition, inverted transposition, and inverted block interchange. In many cases, these repeats keep the ends of rearrangement events unchanged. This suggests that the conservation of ends could possibly be a popular phenomenon in many types of chromosome rearrangement events.

## Additional files

Additional file 1 **Table S3.** Information on Repeats found in *Pseudomonas aeruginosa* strains. **Table S4.** Scaffolds of each of the 25 *Pseudomonas aeruginosa* strains. **Table S5.** Physical positions of repeats and breakpoints of rearrangements in *Pseudomonas aeruginosa* strains. **Table S6.** Scaffolds of each of the 31 *Escherichia coli* Strains. **Table S7.** Physical positions of repeats and breakpoints of rearrangements in *Escherichia coli* strains. **Table S8.** Information on repeats found in the *Escherichia coli* Strains. (XLSX 103 kb)

Additional file 2 Cases where multiple transposition or block interchange events happen between two groups. (DOCX 20 kb)

## Abbreviations

cG: Closest scaffold group
DR: Directed repeat
Indel: Insertion and deletion
*I**n**v*_*d*_: Inversion distance
IR: Inverted repeat
IS: Insertion sequence
kbp: Kilo base pair
mbp: Million base pair
sG: Source scaffold group
