# Supplementary material for: Core-genome scaffold comparison reveals the prevalence that inversion events are associated with pairs of inverted repeats
Source: BMC Genomics. 2017 Mar 29;18:268. doi: 10.1186/s12864-017-3655-0 (PMC5372343; doi:10.1186/s12864-017-3655-0)
Supplement: Supplementary file 2 — Cases where multiple transposition or block interchange events happen between two groups. (DOCX 20 kb) [file 12864_2017_3655_MOESM2_ESM.docx]

**1.** **Pseudomonas aeruginosa, Table 1, From Group 6 to 5:**

G6(strain 16): 1 2 3 4 5 -6 64 -63 -62 -61 -60 -58 -56 -59 -57 -55 54 -53 -52 -50 -51 -49 -48 -47 -46 -45 -44 -43 -42 -41 -40 -39 -38 -37 -36 -35 -34 -32 -33 -31 -30 -29 28 -27 -26 -25 -24 -23 22 -21 -20 19 -18 -17 -16 -15 14 -13 -12 11 -10 -9 8 -7 65 66 68 -67 69

G5(strain 10): 1 2 3 4 5 -6 64 -63 -62 -61 -60 -59 -58 -57 -56 -55 -54 -53 -52 -51 -50 -49 -48 -47 -46 -45 -44 -43 -42 -41 -40 -39 -38 -37 -36 -35 -34 -33 -32 -31 -30 -29 -28 -27 -26 -25 -24 -23 -22 -21 -20 -19 -18 -17 -16 -15 -14 -13 -12 -11 -10 -9 -8 -7 65 66 67 68 69

*There is one more copy of Block -56 and -59.

**Rearrangement distance by our methods: 3+12=15:**

**3 independent transpositions:**

Block -51 (4461bp) swaps with Block -50 (769bp), No IR is found at the breakpoints;

Block -33 (6766bp) swaps with Block -32 (13715bp), No IR is found at the breakpoints;

Inverted transposition: Block -67 (1117bp) swaps with Block 68 (8597bp) and then Block -67 is reversed. No IR is found at the breakpoints.

**After filtering out those independent transpositions, 12 inversions calculated by GRIMM-Synteny:**

======================================================================

Step 0: (Source)

1 2 3 4 5 -6 64 -63 -62 -61 -60 -58 -56 -59 -57 -55 54 -53 -52 -51 -50 -49 -48 -47 -46 -45 -44 -43 -42 -41 -40 -39 -38 -37 -36 -35 -34 -33 -32 -31 -30 -29 28 -27 -26 -25 -24 -23 22 -21 -20 19 -18 -17 -16 -15 14 -13 -12 11 -10 -9 8 -7 65 66 67 68 69

Step 1: -58 through -58: Reversal

1 2 3 4 5 -6 64 -63 -62 -61 -60 58 -56 -59 -57 -55 54 -53 -52 -51 -50 -49 -48 -47 -46 -45 -44 -43 -42 -41 -40 -39 -38 -37 -36 -35 -34 -33 -32 -31 -30 -29 28 -27 -26 -25 -24 -23 22 -21 -20 19 -18 -17 -16 -15 14 -13 -12 11 -10 -9 8 -7 65 66 67 68 69

Step 2: -59 through -59: Reversal

1 2 3 4 5 -6 64 -63 -62 -61 -60 58 -56 59 -57 -55 54 -53 -52 -51 -50 -49 -48 -47 -46 -45 -44 -43 -42 -41 -40 -39 -38 -37 -36 -35 -34 -33 -32 -31 -30 -29 28 -27 -26 -25 -24 -23 22 -21 -20 19 -18 -17 -16 -15 14 -13 -12 11 -10 -9 8 -7 65 66 67 68 69

Step 3: 54 through 54: Reversal

1 2 3 4 5 -6 64 -63 -62 -61 -60 58 -56 59 -57 -55 -54 -53 -52 -51 -50 -49 -48 -47 -46 -45 -44 -43 -42 -41 -40 -39 -38 -37 -36 -35 -34 -33 -32 -31 -30 -29 28 -27 -26 -25 -24 -23 22 -21 -20 19 -18 -17 -16 -15 14 -13 -12 11 -10 -9 8 -7 65 66 67 68 69

Step 4: 28 through 28: Reversal

1 2 3 4 5 -6 64 -63 -62 -61 -60 58 -56 59 -57 -55 -54 -53 -52 -51 -50 -49 -48 -47 -46 -45 -44 -43 -42 -41 -40 -39 -38 -37 -36 -35 -34 -33 -32 -31 -30 -29 -28 -27 -26 -25 -24 -23 22 -21 -20 19 -18 -17 -16 -15 14 -13 -12 11 -10 -9 8 -7 65 66 67 68 69

Step 5: 22 through 22: Reversal

1 2 3 4 5 -6 64 -63 -62 -61 -60 58 -56 59 -57 -55 -54 -53 -52 -51 -50 -49 -48 -47 -46 -45 -44 -43 -42 -41 -40 -39 -38 -37 -36 -35 -34 -33 -32 -31 -30 -29 -28 -27 -26 -25 -24 -23 -22 -21 -20 19 -18 -17 -16 -15 14 -13 -12 11 -10 -9 8 -7 65 66 67 68 69

Step 6: 19 through 19: Reversal

1 2 3 4 5 -6 64 -63 -62 -61 -60 58 -56 59 -57 -55 -54 -53 -52 -51 -50 -49 -48 -47 -46 -45 -44 -43 -42 -41 -40 -39 -38 -37 -36 -35 -34 -33 -32 -31 -30 -29 -28 -27 -26 -25 -24 -23 -22 -21 -20 -19 -18 -17 -16 -15 14 -13 -12 11 -10 -9 8 -7 65 66 67 68 69

Step 7: 14 through 14: Reversal

1 2 3 4 5 -6 64 -63 -62 -61 -60 58 -56 59 -57 -55 -54 -53 -52 -51 -50 -49 -48 -47 -46 -45 -44 -43 -42 -41 -40 -39 -38 -37 -36 -35 -34 -33 -32 -31 -30 -29 -28 -27 -26 -25 -24 -23 -22 -21 -20 -19 -18 -17 -16 -15 -14 -13 -12 11 -10 -9 8 -7 65 66 67 68 69

Step 8: 11 through 11: Reversal

1 2 3 4 5 -6 64 -63 -62 -61 -60 58 -56 59 -57 -55 -54 -53 -52 -51 -50 -49 -48 -47 -46 -45 -44 -43 -42 -41 -40 -39 -38 -37 -36 -35 -34 -33 -32 -31 -30 -29 -28 -27 -26 -25 -24 -23 -22 -21 -20 -19 -18 -17 -16 -15 -14 -13 -12 -11 -10 -9 8 -7 65 66 67 68 69

Step 9: 8 through 8: Reversal

1 2 3 4 5 -6 64 -63 -62 -61 -60 58 -56 59 -57 -55 -54 -53 -52 -51 -50 -49 -48 -47 -46 -45 -44 -43 -42 -41 -40 -39 -38 -37 -36 -35 -34 -33 -32 -31 -30 -29 -28 -27 -26 -25 -24 -23 -22 -21 -20 -19 -18 -17 -16 -15 -14 -13 -12 -11 -10 -9 -8 -7 65 66 67 68 69

Step 10: 58 through 59: Reversal

1 2 3 4 5 -6 64 -63 -62 -61 -60 -59 56 -58 -57 -55 -54 -53 -52 -51 -50 -49 -48 -47 -46 -45 -44 -43 -42 -41 -40 -39 -38 -37 -36 -35 -34 -33 -32 -31 -30 -29 -28 -27 -26 -25 -24 -23 -22 -21 -20 -19 -18 -17 -16 -15 -14 -13 -12 -11 -10 -9 -8 -7 65 66 67 68 69

Step 11: -58 through -57: Reversal

1 2 3 4 5 -6 64 -63 -62 -61 -60 -59 56 57 58 -55 -54 -53 -52 -51 -50 -49 -48 -47 -46 -45 -44 -43 -42 -41 -40 -39 -38 -37 -36 -35 -34 -33 -32 -31 -30 -29 -28 -27 -26 -25 -24 -23 -22 -21 -20 -19 -18 -17 -16 -15 -14 -13 -12 -11 -10 -9 -8 -7 65 66 67 68 69

Step 12: 56 through 58: Reversal (Destination)

1 2 3 4 5 -6 64 -63 -62 -61 -60 -59 -58 -57 -56 -55 -54 -53 -52 -51 -50 -49 -48 -47 -46 -45 -44 -43 -42 -41 -40 -39 -38 -37 -36 -35 -34 -33 -32 -31 -30 -29 -28 -27 -26 -25 -24 -23 -22 -21 -20 -19 -18 -17 -16 -15 -14 -13 -12 -11 -10 -9 -8 -7 65 66 67 68 69

**2. Pseudomonas aeruginosa, Table 1, From Group 8 to 1:**

G8 (strain 18): 1 3 5 6 7 8 9 10 11 12 13 14 15 16 39 17 18 19 20 21 22 23 -48 24 26 2 4 27 28 29 30 31 32 33 34 36 35 37 38 40 -25 41 42 43 44 45 46 47 49 50 51 52 53 54 55 56 57 58 59 60 61 62 63 64 65 66 67 68 69

*There is one more copy of Block 2 and 4.

**Rearrangement distance from G8 to G1 is 4+5=9**

**4 independent transposition:**

Block 39 (2681bp) swaps with Blocks 17~38 in G8. No IR is found at the breakpoints;

Inverted transposition: Block -48 (1253bp) swaps with Blocks 24~47 and then Block -48 is reversed. No IR is found at the breakpoints;

Block 36(4608 bp) swaps with Block 35 (55098bp), No IR is found at the breakpoints;

Inverted transposition: Block -25 (46144bp) swaps with Blocks 26~40 and then Block -25 is reversed. No IR is found at the breakpoints.

**After filtering out those independent transpositions, 5 inversions calculated by GRIMM-Synteny.** ======================================================================

Step 0: (Source)

1 3 5 6 7 8 9 10 11 12 13 14 15 16 17 18 19 20 21 22 23 24 25 26 2 4 27 28 29 30 31 32 33 34 35 36 37 38 39 40 41 42 43 44 45 46 47 48 49 50 51 52 53 54 55 56 57 58 59 60 61 62 63 64 65 66 67 68 69

Step 1: 3 through 3: Reversal

1 -3 5 6 7 8 9 10 11 12 13 14 15 16 17 18 19 20 21 22 23 24 25 26 2 4 27 28 29 30 31 32 33 34 35 36 37 38 39 40 41 42 43 44 45 46 47 48 49 50 51 52 53 54 55 56 57 58 59 60 61 62 63 64 65 66 67 68 69

Step 2: 2 through 2: Reversal

1 -3 5 6 7 8 9 10 11 12 13 14 15 16 17 18 19 20 21 22 23 24 25 26 -2 4 27 28 29 30 31 32 33 34 35 36 37 38 39 40 41 42 43 44 45 46 47 48 49 50 51 52 53 54 55 56 57 58 59 60 61 62 63 64 65 66 67 68 69

Step 3: -3 through -2: Reversal

1 2 -26 -25 -24 -23 -22 -21 -20 -19 -18 -17 -16 -15 -14 -13 -12 -11 -10 -9 -8 -7 -6 -5 3 4 27 28 29 30 31 32 33 34 35 36 37 38 39 40 41 42 43 44 45 46 47 48 49 50 51 52 53 54 55 56 57 58 59 60 61 62 63 64 65 66 67 68 69

Step 4: 3 through 4: Reversal

1 2 -26 -25 -24 -23 -22 -21 -20 -19 -18 -17 -16 -15 -14 -13 -12 -11 -10 -9 -8 -7 -6 -5 -4 -3 27 28 29 30 31 32 33 34 35 36 37 38 39 40 41 42 43 44 45 46 47 48 49 50 51 52 53 54 55 56 57 58 59 60 61 62 63 64 65 66 67 68 69

Step 5: -26 through -3: Reversal (Destination)

1 2 3 4 5 6 7 8 9 10 11 12 13 14 15 16 17 18 19 20 21 22 23 24 25 26 27 28 29 30 31 32 33 34 35 36 37 38 39 40 41 42 43 44 45 46 47 48 49 50 51 52 53 54 55 56 57 58 59 60 61 62 63 64 65 66 67 68 69

**3. E.Coli. Table 2. From G8 to G1:**

G8 (Strain 11): 2 3 4 5 6 8 10 11 -26 12 13 14 15 7 16 17 19 20 22 21 23 24 25 27 28 29 30 32 -46 -45 33 18 34 35 36 37 38 39 41 42 43 44 40 47 -31 48 49 1 9 $

*There are seven copies of Block 45.

**Rearrangement distance is 6+4**

**6 independent transpositions:**

Inverted transposition: Block -26 (24125bp) swaps with Blocks 12~25 and then Block -26 is reversed. No IR is found at the breakpoints;

Block 7 (7035bp) swaps with Blocks 8~15. No IR is found at the breakpoints;

Block 22 (4453bp) swaps with Block 21(83793bp). No IR is found at the breakpoints;

Block 18 (29415bp) swaps with Blocks 19~33. No IR is found at the breakpoints;

Inverted transposition: Block -31 (8437bp) swaps with Blocks 32~47 and then Block -31 is reversed. No IR is found at the breakpoints;

Block 9 (1824bp) swaps with Blocks 2~8 transposition. No IR is found at the breakpoints;

**After filtering out those independent transpositions, 4 inversions calculated by GRIMM-Synteny:**

======================================================================

Step 0: (Source)

2 3 4 5 6 7 8 9 10 11 12 13 14 15 16 17 18 19 20 21 22 23 24 25 26 27 28 29 30 31 32 -46 -45 33 34 35 36 37 38 39 41 42 43 44 40 47 48 49 1

Step 1: 33 through 39: Reversal

2 3 4 5 6 7 8 9 10 11 12 13 14 15 16 17 18 19 20 21 22 23 24 25 26 27 28 29 30 31 32 -46 -45 -39 -38 -37 -36 -35 -34 -33 41 42 43 44 40 47 48 49 1

Step 2: -39 through 44: Reversal

2 3 4 5 6 7 8 9 10 11 12 13 14 15 16 17 18 19 20 21 22 23 24 25 26 27 28 29 30 31 32 -46 -45 -44 -43 -42 -41 33 34 35 36 37 38 39 40 47 48 49 1

Step 3: 33 through 40: Reversal (Repeat exist here)

2 3 4 5 6 7 8 9 10 11 12 13 14 15 16 17 18 19 20 21 22 23 24 25 26 27 28 29 30 31 32 -46 -45 -44 -43 -42 -41 -40 -39 -38 -37 -36 -35 -34 -33 47 48 49 1

Step 4: -46 through -33: Reversal (Destination)

2 3 4 5 6 7 8 9 10 11 12 13 14 15 16 17 18 19 20 21 22 23 24 25 26 27 28 29 30 31 32 33 34 35 36 37 38 39 40 41 42 43 44 45 46 47 48 49 1
